# Coexistence of Nocturia and Frailty and Its Effect on Polypharmacy in Community-Dwelling Older Adults

**DOI:** 10.7759/cureus.84302

**Published:** 2025-05-17

**Authors:** Kyo Takahashi, Tomoki Tanaka, Yasuyo Yoshizawa, Mahiro Fujisaki-Sueda-Sakai, Katsuya Iijima

**Affiliations:** 1 Institute of Gerontology, The University of Tokyo, Tokyo, JPN; 2 Institute of Integrated Research, Institute of Science Tokyo, Tokyo, JPN; 3 Faculty of International Liberal Arts, Juntendo University, Tokyo, JPN; 4 Institute for Future Initiatives, The University of Tokyo, Tokyo, JPN

**Keywords:** frailty, healthy aging, nocturia, older adult, polypharmacy

## Abstract

Introduction: Early interventions for nocturia and frailty are increasingly emphasized to extend healthy life expectancy. These interventions may lead to an increase in the number of drugs administered, potentially resulting in polypharmacy. This study examined the association between nocturia, coexisting frailty, and polypharmacy in community-dwelling older adults.

Methods: We selected 891 older adults without cognitive dysfunction (470 men, 421 women) from a population-based study conducted in 2016 in Kashiwa City, Japan. The association between nocturia, frailty, and polypharmacy was evaluated using sex-stratified logistic regression analysis.

Results: The prevalence of nocturia, frailty, and their coexistence was 56.4%, 6.2%, and 4.0% in men, and 35.2%, 3.8%, and 2.6% in women, respectively. Polypharmacy was observed in 19.4% of men and 13.1% of women. Compared to the group without nocturia or frailty, the adjusted odds ratio (AOR) for polypharmacy was 2.47 (95% confidence interval (CI): 1.29-4.71) in men and 0.99 (95% CI: 0.50-1.95) in women in the group with either nocturia or frailty. The AORs for the group with both nocturia and frailty were 5.33 (95% CI: 1.56-18.17) in men and 1.20 (95% CI: 0.23-6.17) in women.

Conclusion: Polypharmacy is more likely in older men when nocturia and frailty coexist. It is important to ensure that the treatment of nocturia and frailty does not result in a significant increase in the number of medications prescribed.

## Introduction

Nocturia, or frequent nighttime urination, is a prevalent condition in older adults caused by age-related changes, diseases, and medications [1,2]. An international survey of 19,165 participants from five countries found that 71.9% of men and 70.8% of women over 60 years had nocturia [2]. As the number of people with nocturia increases with global aging, it is recognized as a serious problem that can lead to falls, fractures, decreased quality of life, and even death [3-6]. Although conservative methods, such as limiting fluid intake before sleeping, are the first line of treatment, the development of effective drugs is also attracting considerable attention [7].

Frailty prevention is key to healthy aging [8]. Traditionally, long-term care prevention has emphasized addressing age-related diseases such as stroke, arthritis, and dementia [9-11]. However, in addition to preventing such diseases, early interventions aimed at maintaining health in otherwise healthy older adults are increasing [12]. In Japan, which has the highest aging rate worldwide (28.9%), frailty prevention is a cornerstone of national health promotion policy [13]. Like nocturia, conservative methods such as physical exercise are recommended for the treatment of frailty [14]. However, because frailty is associated with various geriatric syndromes, such as depression, falls, and loss of appetite, drug therapy for these symptoms may be prescribed. In recent years, studies have demonstrated a significant association between frailty and multiple drug use due to these accompanying conditions [15].

With increasing attention to nocturia and frailty, concerns have emerged that early intervention may lead to polypharmacy. Polypharmacy, defined as “the concurrent use of multiple medications,” is common in older adults with chronic diseases but can be problematic when it results in adverse drug events [16,17]. Recent studies have found various negative effects of polypharmacy in older adults, including potentially severe drug interactions, emergency admissions, new-onset sarcopenia, and poor physical performance [18-21]. Although studies have shown an association between nocturia and frailty [22], the impact of their coexistence on polypharmacy has not been clarified. This cross-sectional study examined whether polypharmacy significantly increases when nocturia and frailty coexist in community-dwelling older adults.

## Materials and methods

Study design and participants

**Figure 1:**
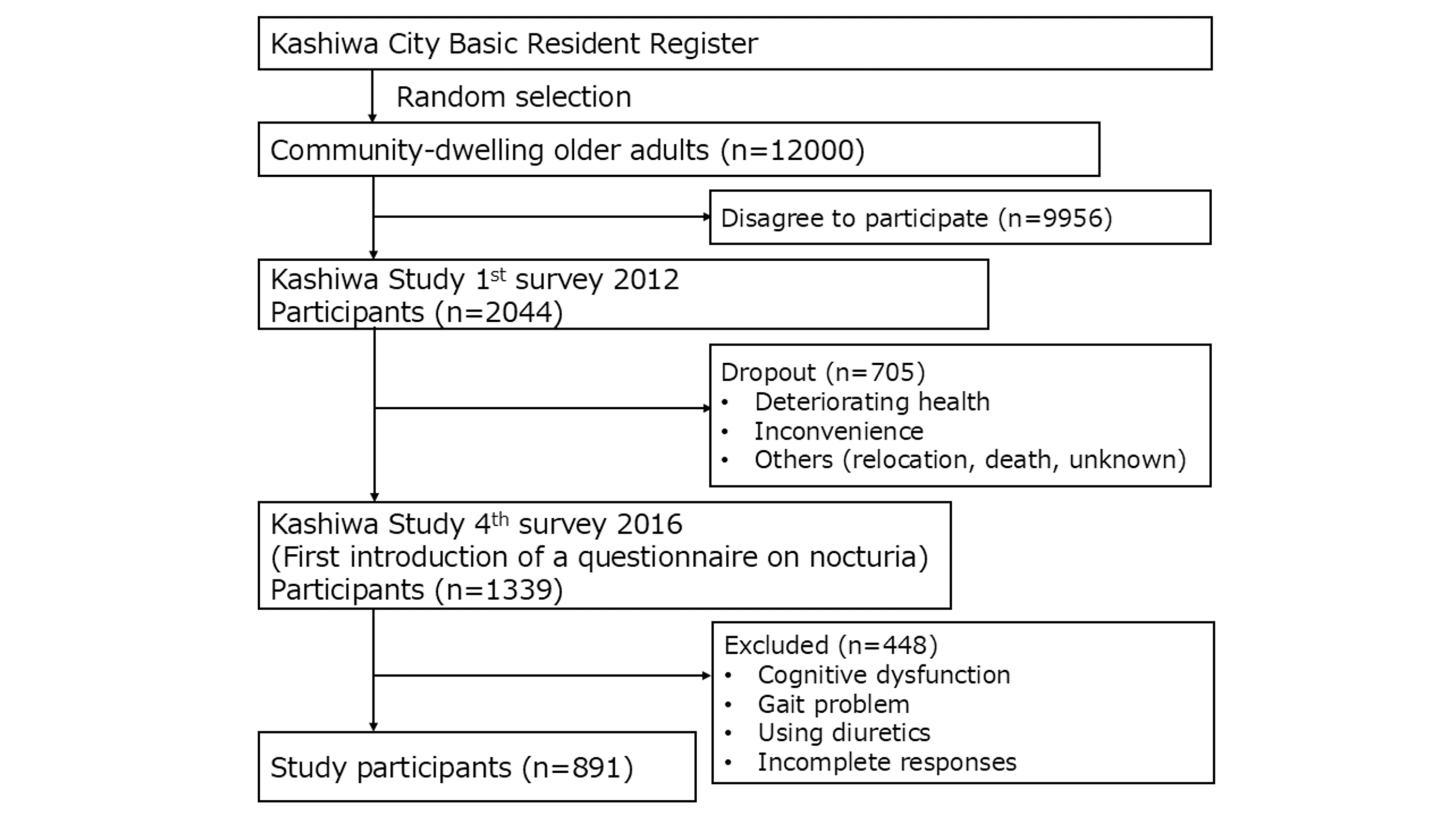
Participant selection process

We selected 891 community-dwelling older adults from a population-based study conducted in Kashiwa City, Japan (the Kashiwa Study). The Kashiwa Study is a cohort study that began in 2012 with the aim of generating evidence to support health promotion in older adults [20,23]. In 2012, 12,000 community-dwelling older adults aged 65 years or older were randomly selected from the Kashiwa City Basic Resident Register and invited to participate. Of these, 2,044 agreed to participate and completed a baseline survey. The current analysis targeted 1,339 individuals who took part in the fourth survey, conducted from 06 September to 27 October 2016, when nocturia was first assessed. Participants with cognitive dysfunction (Mini-Mental State Examination score <24), mobility disability (any problems while walking), those using diuretics (loop diuretics, thiazides, or sodium-glucose cotransporter 2 inhibitors), or those with incomplete responses to essential items were excluded (Figure 1).

Measurements

Nocturia

Nocturia was assessed using the self-reported question, "How many times did you get up to urinate after you went to bed and before you got up in the morning?" Of the six possible answers (0, 1, 2, 3, 4, or more times), participants who answered “two or more times” were considered to have nocturia, based on previous studies [1,6].

Frailty

Frailty was measured using the Japanese version of the Cardiovascular Health Study (J-CHS) criteria [24,25]. The J-CHS criteria included five components: shrinking (Have you unintentionally lost 2 kg or more in the past 6 months?), low activity (Do you engage in moderate levels of physical exercise or sports aimed at health? Do you engage in low levels of physical exercise aimed at health?), exhaustion (In the past 2 weeks, have you felt tired without a reason?), weakness (grip strength <28 kg in men or <18 kg in women), and slowness (gait speed <1.0 m/s). Shrinking, low activity, and exhaustion were assessed via self-administered questionnaires, whereas weakness and slowness were measured using a Smedley-type grip strength meter and a 5-meter walking test, respectively. Participants who met three or more of the five criteria were considered frail.

Polypharmacy

Polypharmacy is defined as the simultaneous intake of multiple drugs (five to six or more) [16,19]. Based on a previous study analyzing the association between the number of drug types and adverse drug reactions in older patients in Japan, polypharmacy was defined as the daily intake of six or more drug types [26]. Participants reported the types and number of medications they regularly took, referring to their medication records. Topical or over-the-counter medications were excluded. Trained nurses reviewed the medication records with participants to ensure accuracy.

Statistical analysis

Descriptive data were reviewed separately for men and women. To analyze the impact of overlapping nocturia and frailty on polypharmacy, participants were divided into three groups: (1) neither nocturia nor frailty, (2) either nocturia or frailty, and (3) both nocturia and frailty. Sex-stratified logistic regression analysis was performed using the first group as a reference. Covariates included a history of non-communicable diseases, such as heart disease, cancer, diabetes, chronic kidney disease, hypertension, and dyslipidemia. As a sensitivity analysis, an age-adjusted, sex-stratified logistic regression was conducted among participants with hypertension only. All statistical analyses were conducted using IBM SPSS Statistics for Windows, Version 27 (Released 2020; IBM Corp., Armonk, New York).

Ethical approval

The study was conducted in accordance with the principles of the Declaration of Helsinki. The Research Ethics Committee of the University of Tokyo approved all study procedures (approval number: 24-229 (change to 12-8), approval date: July 23, 2024). Using a research explanation document, we informed each participant about important matters such as the study outline, voluntary participation, freedom of withdrawal, and protection of personal information. All participants provided written informed consent.

## Results

Table 1 presents the characteristics of the 891 participants (470 men, 421 women). The mean age was 76.2 years (standard deviation: 5.1); male participants were slightly more (52.7%) than female participants (47.3%). The prevalence of nocturia and frailty in men was 56.4% and 6.2%, respectively, with a coexistence rate of 4.0%. In women, the percentages were 35.2%, 3.8%, and 2.6%, respectively. Polypharmacy was observed in 19.4% of men and 13.1% of women.

**Table 1:** Characteristics of the participants n: number, SD: standard deviation.

Characteristics	Total		Men		Women	
	n (N = 891)	%	n (N = 470)	%	n (N= 421)	%
Age (mean ± SD)	76.2 ± 5.1		76.5 ± 5.2		75.9 ± 4.9	
Nocturia	413	46.4	265	56.4	148	35.2
Frailty	45	5.1	29	6.2	16	3.8
Coexistence						
Neither nocturia nor frailty	463	52.0	195	41.5	268	63.7
Either nocturia or frailty	398	44.7	256	54.5	142	33.7
Both nocturia and frailty	30	3.4	19	4.0	11	2.6
Polypharmacy	146	16.4	91	19.4	55	13.1
History of non-communicable diseases						
Heart disease	119	13.4	84	17.9	35	8.3
Cancer	154	17.3	101	21.5	53	12.6
Diabetes	96	10.8	66	14.0	30	7.1
Chronic kidney disease	11	1.2	6	1.3	5	1.2
Hypertension	395	44.3	232	49.4	163	38.7
Dyslipidemia	296	32.2	121	25.7	175	41.6

**Figure 2:**
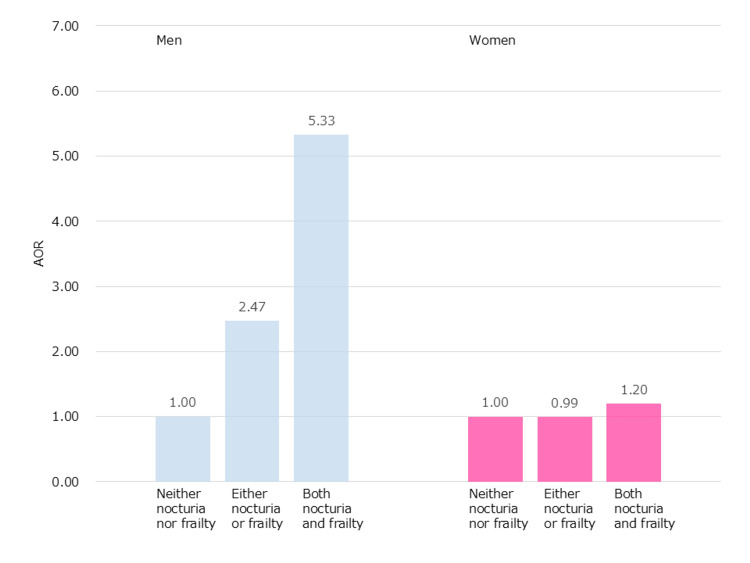
Different odds ratios among the three groups by sex The “neither nocturia nor frailty” group was used as a reference. Age and history of non-communicable diseases (heart disease, cancer, diabetes, chronic kidney disease, hypertension, and dyslipidemia) were entered as covariates into each logistic regression analysis.

Figure 2 shows the results of the logistic regression analysis. When the group without nocturia and frailty was used as the reference, the adjusted odds ratio (AOR) for polypharmacy was 2.47 (95% confidence interval (CI): 1.29-4.71) in men and 0.99 (95% CI: 0.50-1.95) in women in the group with either nocturia or frailty. The AORs in the nocturia and frailty coexistence groups were 5.33 (95% CI: 1.56-18.17) in men and 1.20 (95% CI: 0.23-6.17) in women.

The results of the sensitivity analysis are shown in Table 2. The AOR for polypharmacy was 3.40 (95% CI: 1.48-7.79) in men and 1.29 (95% CI: 0.57-2.93) in women in the group with either nocturia or frailty. The AORs in the nocturia and frailty coexistence groups were 10.86 (95% CI: 2.60-45.35) in men and 0.92 (95% CI: 0.12-7.10) in women.

**Table 2:** Results of the logistic regression analysis predicting polypharmacy among participants with hypertension The "neither nocturia nor frailty" group was used as a reference. Age was entered as a covariate into each logistic regression analysis.

Stratified variables	Adjusted odds ratio (95% CI)		
	Neither nocturia nor frailty	Either nocturia or frailty	Both nocturia and frailty
Men	1.00	3.40 (1.48-7.79)	10.86 (2.60-45.35)
Women	1.00	1.29 (0.57-2.93)	0.92 (0.12-7.10)

## Discussion

Polypharmacy in older adults is a serious problem, and clarifying the attributes that predispose them to this condition is important. This study found that community-dwelling older men with both nocturia and frailty were 5.33 times more likely to have polypharmacy than those without nocturia and frailty. Although both nocturia and frailty can be partially managed through conservative methods, having both simultaneously may increase health anxiety, resulting in more visits to medical facilities. Kojima et al. found that increased visits were a risk factor for polypharmacy in nursing home residents [27]. Specialized treatments for nocturia and frailty are often provided by different medical departments. Frail individuals with nocturia may require multiple medications and visit different medical facilities, potentially increasing the risk of polypharmacy and prescription cascade, eventually leading to potentially inappropriate medication use.

The presence of male-specific diseases such as benign prostatic hyperplasia (BPH) may explain why a significant difference was found only in men. While overactive bladder and BPH are both well-known causes of nocturia, BPH is unique to men. Drugs such as α1 adrenergic receptor blockers or phosphodiesterase 5 inhibitors are often used to treat BPH before considering surgery [28-30]. In addition to these drugs, drugs used for nocturia and frailty-related symptoms may also be administered, leading to polypharmacy in older men. Given this background, further attention should be paid to polypharmacy in older men with nocturia.

Besides BPH, many other diseases can affect nocturia, frailty, and polypharmacy. For example, hypertension is a cause of nocturia and is related to an increase in medication [30]. In this study, we performed a logistic regression analysis with various non-communicable diseases, including hypertension, as covariates, and also conducted a sensitivity analysis that included only participants with hypertension. In the sensitivity analysis, the results showed that, compared with the "neither nocturia nor frailty" group, the "either nocturia or frailty" group was 3.40 times as likely to have polypharmacy, and the "both nocturia and frailty" group was 10.86 times as likely to have polypharmacy in men. This result was consistent with the results of all participants, demonstrating the robustness of the analysis results.

Drug treatment is certainly effective for both nocturia and frailty, particularly in patients with severe symptoms who cannot recover through self-care. However, when both conditions coexist, thorough checking that the same type of medication is not prescribed twice and preventing a prescription cascade is necessary. Visits to multiple medical facilities are common in aging societies. Novel systems being developed and implemented are expected to visualize the status of everyone’s drug treatment and strengthen the confirmation function at each medical facility. Moreover, the promotion of conservative self-care methods should be considered before initiating drug treatment. For example, daily exercise can help prevent and improve nocturia and frailty. By promoting the prevention and improvement of nocturia and frailty in a comprehensive manner, it is expected that the awareness and behavior of many older adults will change, bringing us closer to realizing a society in which people can lead long and healthy lives.

This study has some limitations. First, the causal relationship between nocturia, frailty, and polypharmacy remains unknown owing to the cross-sectional study design. Although it is assumed that the number of drugs increased because of the combination of nocturia and frailty, it cannot be denied that the symptoms of nocturia and frailty may have been induced as a result of using a combination of multiple drugs. Second, because a self-administered questionnaire was used, recall bias may have been present. To minimize the influence of this bias, we asked the participants to refer to their medication notebooks to record the number of drugs they were taking and check the content of the questionnaire face-to-face before submitting it. Third, polypharmacy was defined only by the number of drugs, without considering other factors like the type of drug used, adherence, and underlying disease. Although we excluded those using diuretics, which directly affect polypharmacy and nocturia, future studies on polypharmacy should adopt a design that can adjust various characteristics of drugs when treating nocturia as a main variable.

## Conclusions

Despite the above limitations, this study is significant as the first, to our knowledge, to demonstrate that the coexistence of nocturia and frailty may cause polypharmacy. It is important to ensure that the treatment of nocturia and frailty in the early stages does not lead to an unnecessary increase in the number of drugs administered. To ensure that early treatment of nocturia and frailty does not lead to polypharmacy, further research to clarify causal relationships and awareness-raising activities to change society should be conducted.
